# 25-Hydroxyvitamin D status does not affect energy metabolism among young, healthy, non-obese adults: a metabolic chamber study

**DOI:** 10.3389/fendo.2024.1501818

**Published:** 2024-11-18

**Authors:** Lin Zhang, Haogang Cai, Xiaorong Bai, Wensheng Xiao, Zhen-Bo Cao, Yang Zhang

**Affiliations:** ^1^ Institute of Physical Education, Jiangsu Normal University, Xuzhou, China; ^2^ School of Physical Education, Shangqiu Normal University, Shangqiu, China; ^3^ School of Physical Education, Huzhou University, Huzhou, China; ^4^ College of Physical Education, Hunan Normal University, Changsha, China; ^5^ School of Exercise and Health, Shanghai University of Sport, Shanghai, China; ^6^ Independent person, Windermere, FL, United States; ^7^ Independent Researcher, Dakar, Senegal

**Keywords:** energy expenditure, lipid profile, oxidation rate, vitamin D, obesity, physical activity

## Abstract

**Purpose:**

here is a general consensus that an inverse relationship exists between vitamin D status and body mass index (BMI) in overweight and obese individuals, leading to the hypothesis that vitamin D deficiency may contribute to the development of unfavorable metabolic phenotypes. However, evidence from non-obese adults remains limited. This study measured energy metabolism in non-obese adults using a metabolic chamber and explored its association with vitamin D status.

**Methods:**

Sixty-nine healthy adults (mean age = 22.8 years, mean BMI = 20.7 kg/m^2^) participated in this cross-sectional study. Participants were categorized into vitamin D-deficient, insufficient, and sufficient groups based on the Chinese classification for total 25(OH)D levels (WS/T 677–2020). They performed typical daily activities in a metabolic chamber, where their baseline lipid profile, 24-hour energy expenditure, and substrate oxidation were measured.

**Results:**

A two-way ANOVA (seasonality × 25(OH)D) revealed no statistically significant differences in total energy expenditure, resting energy expenditure, sleeping energy expenditure, walking energy expenditure, carbohydrate oxidation rate, or fat oxidation rate among the three groups (p > 0.05). These results remained consistent even after adjusting for fat-free mass. Although statistically significant correlations were found between 25(OH)D status and certain lipid profile markers (i.e., total cholesterol, high-density lipoprotein, and free fatty acid) (p < 0.05), these correlations were weak, with Pearson’s correlation coefficients below 0.3.

**Conclusions:**

Total 25(OH)D status does not affect energy metabolism in young, healthy, non-obese adults. Along with existing evidence, this suggests that low 25(OH)D status is more likely a consequence of unfavorable metabolic phenotypes rather than a contributing factor.

**Clinical trial registration:**

https://www.chictr.org.cn, identifier ChiCTR-IIR-17010604.

## 1 Introduction

Vitamin D, a fat-soluble sterol compound and hormone precursor, plays a critical role in various physiological functions (1). Among these, its involvement in human energy metabolism has garnered significant attention over the past decade (2). Research has shown that vitamin D status is a positive predictor of resting energy expenditure in adults, independent of obesity or metabolic syndrome (3). This has led to the hypothesis that vitamin D insufficiency or deficiency may be an independent risk factor for unfavorable metabolic phenotypes such as obesity, type 2 diabetes, and dyslipidemia — primarily supported by observational studies (4). In the Framingham Heart Study, for example, vitamin D status was inversely associated with waist circumference and visceral adipose tissue in non-diabetic individuals (5). Similar associations have been observed across various populations, including obese adults (6), healthy children (7), and older adults (8). Given this body of evidence, vitamin D supplementation has been explored as a potential strategy to improve metabolic health. However, many intervention studies have not demonstrated a causal effect of vitamin D status on obesity metrics or weight loss (9, 10). Despite this, the prevalence of poor vitamin D status among individuals with metabolic diseases highlights the need for further research into the relationship between vitamin D and energy metabolism.

There are three main limitations in research on vitamin D’s role in energy metabolism. First, while rodent models often provide valuable insights into human disease development and treatments, the effects of vitamin D metabolism on energy homeostasis differ significantly between animal and human studies (11). Given this limitation, more rigorously controlled experimental studies in humans are necessary. Second, most studies in this field rely on metabolic cart measurements to estimate whole-body energy metabolism (3, 12). Methodologically, metabolic cart-based assessments of energy expenditure and macronutrient oxidation, especially through short-term measurements, may yield inaccurate results (13). In contrast, metabolic chambers, which allow unrestricted human movement and locomotion, provide not only more accurate measurements but also a better representation of real-life energy metabolism. Yet, the high costs and demanding research conditions (e.g., 24-hour observation) associated with metabolic chambers pose significant challenges. To our knowledge, studies in this area using metabolic chambers are scarce. Given the sensitivity needed to detect small yet meaningful effects of vitamin D on physiological functions, more studies utilizing metabolic chambers are required. Third, vitamin D deficiency and essential mineral deficiencies in general are common worldwide (14), affecting not only obese populations but also healthy individuals. From a preventive medicine perspective, understanding vitamin D’s role in energy metabolism among healthy individuals is of significant public health interest. In vitamin D-sufficient young adults, for example, additional vitamin D supplementation has been shown to have no effect on resting energy expenditure (12). As outlined above, due to the limited data from representative 24-hour energy metabolism studies, there are currently no conclusive guidelines to determine vitamin D requirements for metabolic health in the general population. Specifically, data on young individuals with vitamin D insufficiency or deficiency remain scarce.

Therefore, the purpose of this study was to explore the impact of vitamin D status, measured by 25-hydroxyvitamin D (25(OH)D), on energy expenditure and substrate oxidation in young, healthy, non-obese adults. To address the limitations of previous research, a metabolic chamber was utilized to measure energy metabolism over a 24-hour period during typical daily activities. The findings are expected to provide new insights into the effects of vitamin D status on metabolic health in the general population.

## 2 Methods

### 2.1 Study design

This trial (clinical registration: ChiCTR-IIR-17010604) was approved by the Ethics Committee of the Shanghai University of Sport (approval # 2015009). According to China’s guidelines for vitamin D deficiency screening (Chinese Standard GB/T: WS/T 677–2020), vitamin D status is classified as follows: adequate (serum total 25(OH)D ≥ 50 nmol/L), insufficient (30 ≤ 25(OH)D < 50 nmol/L), and deficient (25(OH)D < 30 nmol/L). This study aimed to use a one-way ANOVA to examine the relationship between 25(OH)D status and energy metabolism. Assuming a Cohen’s d of 0.5, p-value of 0.05, and 80% power for F-tests, the minimum required sample size for statistical significance in this cross-sectional, three-group design was 14 participants per group.

Potential participants were recruited from the Shanghai University of Sport between March 2017 and October 2018. Eligibility criteria included: age between 18 and 30 years, body mass index (BMI) ≤ 25 kg/m², and no use of medications or nutritional supplements (e.g., vitamin B_2_, vitamin D, or weight-loss drugs) that could influence vitamin D status, energy metabolism, or lipid profile within the six months before the study. All participants provided written informed consent. A total of 69 students, most of whom were female, were recruited. Their general characteristics are presented in **Table 1**. Apart from differences in 25(OH)D status and maximal oxygen consumption, no significant differences were observed among the three groups.

**Table 1:** Demographics characteristics.

Demographics	Serum 25(OH)D status			Pairwise comparison
	Deficiency	Insufficiency	Adequacy	
n (men)	30 (3)	25 <<(>>2<<)>>	14 (3)	–
25(OH)D (nmol·L^-1^)	22.1 ± 5.6	37.4 ± 4.1	57.1 ± 7.1	*F* = 200, *p* < 0.001
Age (years)	23.6 ± 2.0	22.3 ± 2.1	22.2 ± 2.5	*F* = 3.2, *p* = 0.048
Height (cm)	161 ± 6	162 ± 8	166 ± 9	*F* = 2.9, *p* = 0.063
Body weight (kg)	53.8 ± 6.1	54.4 ± 9.8	57.4 ± 7.5	*F* = 1.0, *p* = 0.366
BMI (kg·m^2^)	20.8 ± 1.7	20.5 ± 2.5	21.0 ± 2.4	*F* = 0.2, *p* = 0.826
Fat free mass (kg)	36.1 (5.4)	36.9 (3.9)	38.4 (8.1)	χ2 = 1.5, *p* = 0.461
Fat (%)	29.3 ± 6.3	28.5 ± 7.0	27.5 ± 7.4	*F* = 0.3, *p* = 0.708
Body surface area^a^	1976 ± 179	1984 ± 248	2105 ± 270	*F* = 1.7, *p* = 0.183
VO_2max_ (ml·kg^-1^·min^-1^)	30.6 ± 5.1	34.2 ± 4.4	36.4 ± 6.1	*F* = 6.6, *p* = 0.003
Parathyroid hormone (pg·ml^-1^)	48.5 ± 16.8	58.3 ± 23.5	64.2 ± 29.5	*F* = 2.7, *p* = 0.077

Data are expressed as mean ± standard deviation or median (interquartile range). ^a^based on the Du Bois formula.

### 2.2 Protocol

At least one week before the metabolic chamber test, participants completed a maximum oxygen consumption test using a cycle ergometer and the Cosmed K4b2 portable metabolic system (Cosmed, Inc., Rome, Italy). During the same visit, body composition was assessed using a dual-energy X-ray absorptiometry scanner (GE Lunar Prodigy, USA).

On the experimental day, blood samples were collected from participants at 7:30 AM by accredited nurses following a 12-hour overnight fast. After resting at room temperature for 20–30 minutes, the samples were centrifuged at 3000 rpm for 15 minutes at 4°C. The serum was then transferred to additive-free tubes and immediately stored at -80°C for further analysis. Serum 25(OH)D concentrations were measured using the 25-Hydroxy Vitamin D EIA (AC-57SF1, IDS Ltd), which has a sensitivity of 3.3 nmol/L and intra- and inter-assay coefficients of variation of 8.9% and 10.6%, respectively. Immunoreactive parathyroid hormone levels were determined using the EIA-4140 (DRG Inc.), with intra- and inter-assay coefficients of variation of 9.6%. Serum total cholesterol was measured using the cholesterol oxidase method.

Participants’ 24-hour energy metabolism was measured in an indirect calorimetry chamber (Fuji High Accuracy Human Calorimeter, FHC-20S, Japan), which was maintained at 25°C with 50% humidity. Participants followed a structured activity protocol, as outlined in **Table 2**, from 10 AM on day 1 to 10 AM on day 2. Three meals were provided based on age and body mass, adhering to the estimated energy requirements outlined in the Chinese Dietary Reference Intakes (Chinese Standard GB/T: WS/T 578.1–2017). The macronutrient composition of the meals was standardized to provide 50–60% of total calories from carbohydrates, 20–30% from fat, and 15–20% from protein. Breakfast, lunch, and dinner accounted for 30%, 40%, and 30% of the total daily caloric intake, respectively. Using minute-by-minute data on oxygen consumption and carbon dioxide production, 24-hour energy expenditure was calculated with Weir’s formula (15). The Frayn equation was applied to estimate carbohydrate and fat oxidation rates (16).

**Table 2:** Schedule of 2-day activities in the metabolic chamber.

Day 1	Activity of day 1	18:00 PM	Viewing smart phone at a desk
9:30 AM	Enter chamber	18:30 PM	Writing at a desk
10:00 AM	Working on a laptop	19:00 PM	listening to music
11:00 AM	Doing a cervical and lumbar gymnastics	19:30 PM	Reading at a desk
11:30 AM	Viewing smart phone at a desk	20:00 PM	Watching video
12:00 PM	Lunch	22:00 PM	Quietly resting on bed (rest metabolic rate)
12:20 PM	Doing housework	22:45 PM	Washing and ready to sleep
12:30 PM	Midday break	23:00 PM	Sleep
13:30 PM	Listening to music	Day 2	Activity of day 2
14:00 PM	Reading at a desk	7:00 AM	Going to the bathroom
14:30 PM	Writing at a desk	7:15 AM	Resting on bed (basal metabolic rate)
15:00 PM	Doing a cervical and lumbar gymnastics	8:00 AM	Breakfast
15:30 PM	Watching video	8:30 AM	Viewing smart phone at a desk
16:30 PM	Treadmill walking at 3.2 km/h	9:00 AM	Treadmill walking at 5.6 km/h
16:40 PM	Working on a laptop	9:30 AM	Reading at a desk
17:40 PM	Diner	10:00 AM	Energy metabolism collection ends

### 2.3 Statistics

**Figure 1:**
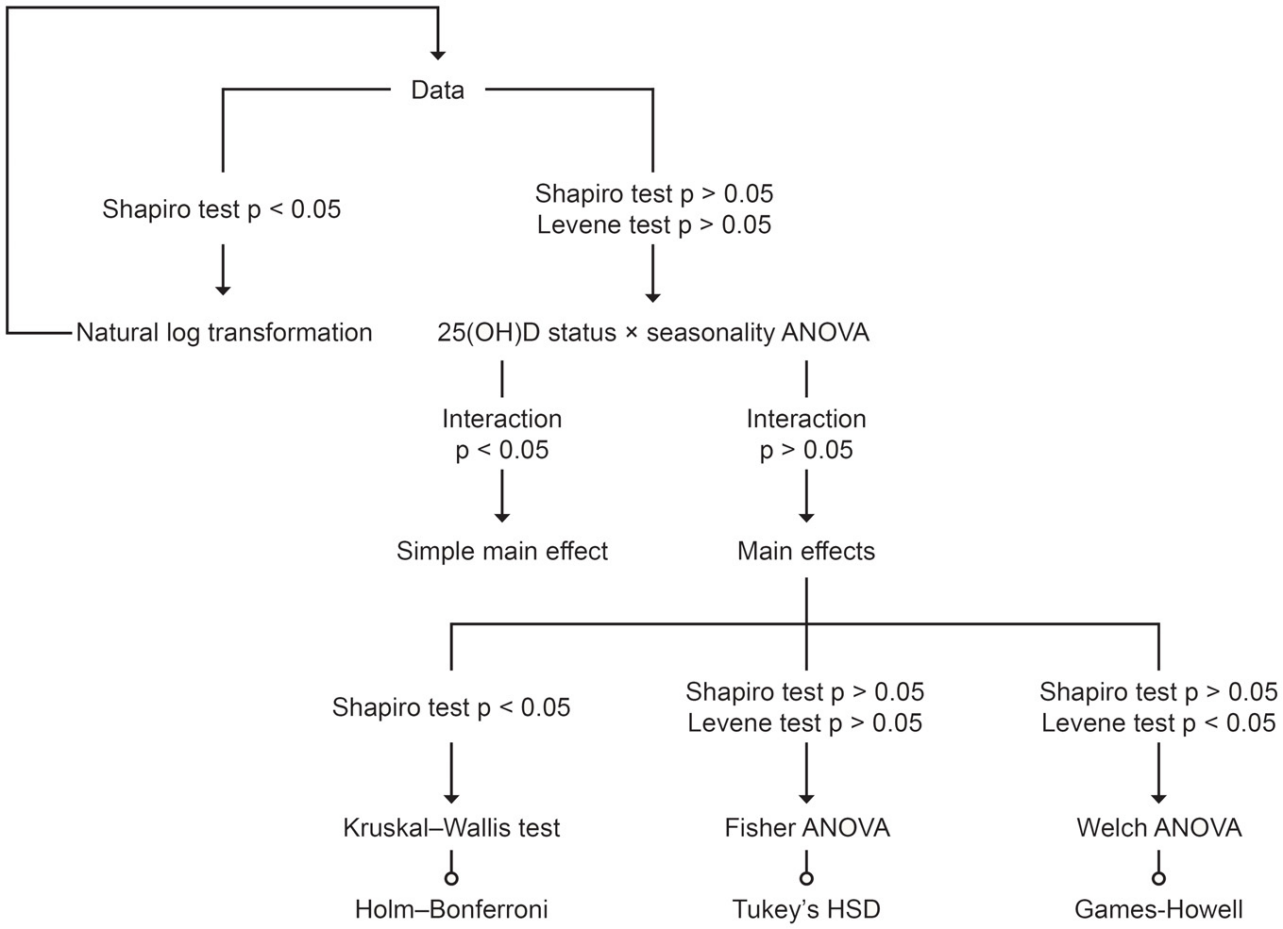
Decision tree of two-way ANOVA.

The de-identified data supporting the conclusions of this study are deposited at Figshare (DOI: 10.6084/m9.figshare.27280311.v1). All statistical analyses were performed using R version 4.4.1 (Race for Your Life). Considering the seasonal effects on the body’s vitamin D production (17), a two-way ANOVA, as illustrated in **Figure 1**, was used to examine the relationship between 25(OH)D status and energy metabolism. Additionally, Pearson’s correlation coefficient or Spearman’s rank correlation coefficient was employed to assess the relationship between 25(OH)D status and the lipid profile. To address asymmetry due to differing measurement units, log transformations were applied before conducting the correlation analysis. Statistical significance was set at the 5% level.

## 3 Results

**Figure 2:**
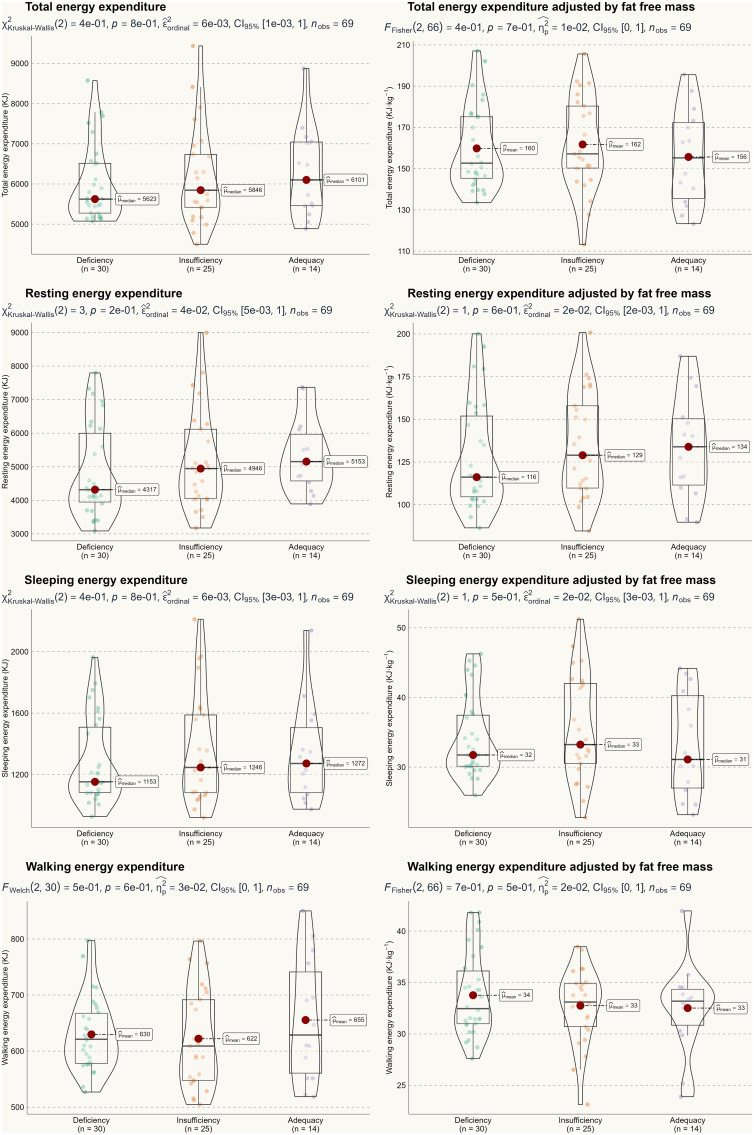
Energy metabolism by serum 25(OH)D status.

**Figure 3:**
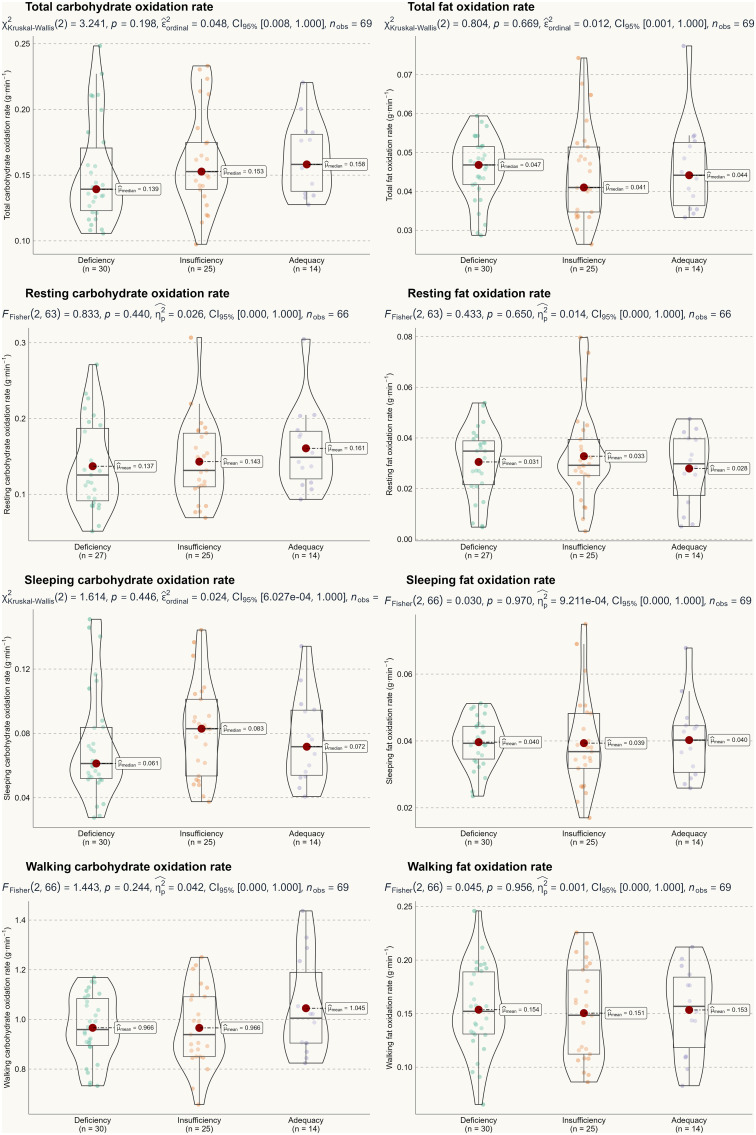
Substrate oxidation rate by serum 25(OH)D status.

Based on the two-way ANOVA, no significant interaction effect was detected, with F-test values ranging from 0.22 to 2.52 and p-values between 0.22 and 0.95. Therefore, we can conclude that seasonality did not influence the primary outcomes of this study. A one-way ANOVA was used to assess the main effect of 25(OH)D status, and **Figures 2** and **3** illustrate energy metabolism across the three groups. In summary, neither energy expenditure nor substrate oxidation rates were significantly affected by 25(OH)D status, even after adjusting energy metabolism for fat-free mass (as shown in **Figure 2**), body weight (not shown here), or muscle mass (not shown here). The main effect of seasonality is detailed in **Table 3**, where participants tested in the spring exhibited significantly higher values across most energy metabolism metrics.

**Table 3:** Results of seasonality effect.

Variable	ANOVA	Spring	Summer	Autumn
TEE (KJ)	*F*_Welch_(2, 40.5) = 31.9, *p* < 0.001	7184 ± 1002	5629 ± 666^***^	5436 ± 317^###^
REE (KJ)	*F*_Welch_(2, 42.5) = 33.1, *p* < 0.001	6454 ± 1229	4415 ± 736^***^	4175 ± 553^###^
SEE (KJ)	*F*_Welch_(2, 39.4) = 41.2, *p* < 0.001	1663 ± 272	1136 ± 153^***^	1140 ± 69^###^
WEE (KJ)	*F*_Fisher_ *(*2, 66*)* = 4.6, *p* = 0.014	671 ± 91	614 ± 77^*^	608 ± 63^#^
TCOR (g·min^-1^)	*F*_Welch_(2, 42.7) = 35.6, *p* < 0.001	0.194 ± 0.031	0.143 ± 0.021^***^	0.130 ± 0.017^###^
TFOR (g·min^-1^)	*F*_Fisher_ *(*2, 66*)* = 4.6, *p* = 0.03	0.050 ± 0.013	0.042 ± 0.009^*^	0.044 ± 0.007
RCOR (g·min^-1^)	*F*_Fisher_ *(*2, 63*)* = 24.7, *p* < 0.001	0.194 ± 0.056	0.122 ± 0.033^***^	0.111 ± 0.033^###^
RFOR (g·min^-1^)	*F*_Welch_(2, 40.1) = 1.0, *p* = 0.4	0.035 ± 0.022	0.028 ± 0.012	0.030 ± 0.010
SCOR (g·min^-1^)	*F*_Fisher_ *(*2, 66*)* = 33.3, *p* < 0.001	0.106 ± 0.028	0.060 ± 0.018^***^	0.061 ± 0.018^###^
SFOR (g·min^-1^)	*F*_Fisher_(2, 66) = 5.8, *p* = 0.005	0.045 ± 0.013	0.036 ± 0.010^**^	0.037 ± 0.007^#^
WCOR (g·min^-1^)	*F*_Fisher_(2, 66) = 5.2, *p* = 0.008	1.050 ± 0.171	0.974 ± 0.133	0.906 ± 0.133^##^
WFOR (g·min^-1^)	*F*_Fisher_(2, 66) = 1.8, *p* = 0.2	0.161 ± 0.041	0.141 ± 0.039	0.157 ± 0.041

Data are expressed as mean ± standard deviation. TEE, total energy expenditure; REE, resting energy expenditure; SEE, sleeping energy expenditure; WEE, walking energy expenditure; TCOR, total carbohydrate oxidation rate; TFOR, total fat oxidation rate; RCOR, resting carbohydrate oxidation rate; RFOR, resting fat oxidation rate; SCOR, sleeping carbohydrate oxidation rate; SFOR, sleeping fat oxidation rate; WCOR, walking carbohydrate oxidation rate; WFOR, walking fat oxidation rate. ^*^ *p* < 0.05 for spring vs. summer; ^#^ *p* < 0.05 for spring vs. autumn; ^**^ *p* < 0.01 for spring vs. summer; ^##^ *p* < 0.01 for spring vs. autumn; ^***^ *p* < 0.001 for spring vs. summer; ^###^ *p* < 0.001 for spring vs. autumn. Excluding the estimated RCOR and RFOR, the sample sizes for spring, summer, and autumn were 24, 26, and 19, respectively. For the estimated RCOR and RFOR, we lost measurements from one participant in each.

**Table 4** summarizes the correlations between 25(OH)D status and the lipid profile. Although statistically significant relationships were observed for total cholesterol, high-density lipoprotein, and free fatty acid, these correlations were weak.

**Table 4:** Correlations between the log transformed serum 25(OH)D (explanatory variable) and lipid profile (response variable).

Response variable	Statistics
ln(Total cholesterol)	*t*(67) = -2.33, *p* = 0.02, *r* = -0.27, CI_95%_ = -0.48 to -0.04
ln(Low-density lipoprotein)	*S* = 62311, *p* = 0.26, *ρ* = -0.14, CI_95%_ = -0.37 to 0.11
ln(High-density lipoprotein)	*t*(67) = -2.50, *p* = 0.02, *r* = -0.29, CI_95%_ = -0.49 to -0.06
ln(Triglycerides)	*S* = 62655, *p* = 0.24, *ρ* = -0.14, CI_95%_ = -0.37 to 0.10
ln(Free fatty acid)	*t*(65) = -2.34, *p* = 0.02, *r* = -0.28, CI_95%_ = -0.49 to -0.04

## 4 Discussion

In a group of young, healthy, non-obese adults, we demonstrate that total 25(OH)D status does not influence energy expenditure or lipid profiles typically associated with overweight and obesity. A novel aspect of this study is that the findings are based on 24-hour continuous measurements of typical human activities conducted in a metabolic chamber. The comprehensiveness of this data set provides new insights into the role of vitamin D in human energy metabolism.

In rodent models, vitamin D receptor-null or vitamin D-deficient mice exhibit an enhanced rate of fatty acid β-oxidation in white adipose tissue and reduced lipid accumulation in the liver, both of which are beneficial for weight management (18, 19). However, it is now clear that these results are not transferable to humans. A substantial body of literature demonstrates an inverse relationship between circulating 25(OH)D levels and fat mass in children (20), adults (21), and even athletes (22). Emerging cellular evidence suggests a potential causal relationship (23), leading to new hypotheses that propose vitamin D status could serve as an adjuvant therapy for unfavorable metabolic phenotypes (24, 25). This existing research prompted the present study, which aimed to clarify the relationship between vitamin D status and human energy metabolism. However, none of the key metrics — energy expenditure or substrate oxidation — differed among non-obese adults with deficient, insufficient, or sufficient 25(OH)D status. Similarly, the significant yet weak correlations between 25(OH)D status and lipid profile do not provide conclusive evidence regarding vitamin D’s role in body weight regulation. To our knowledge, this is the first study to report negative evidence under controlled metabolic chamber conditions.

Despite the well-established inverse association between 25(OH)D status and obesity, our findings suggest that low vitamin D status may not be the cause of unfavorable metabolic phenotypes but rather a consequence. This helps explain the conflicting results between observational and intervention studies (9). Several plausible explanations support this notion. First, aside from dietary sources and supplementation, sun exposure is the main contributor to endogenous cholecalciferol production in the skin. Overweight and obese individuals, often due to low mobility and motivation, engage in less outdoor physical activity (26, 27), which limits natural vitamin D synthesis. Second, overweight and obesity disrupt adipose tissue homeostasis and induce hepatic dysfunction, both of which can affect vitamin D metabolism. In obese individuals, the expression of the cytochrome P450 gene family — the primary 25(OH)D hydroxylase — is down-regulated (28, 29), interfering with downstream circulating 25(OH)D activity. Evidence from obese patients undergoing gastric bypass surgery demonstrates that obesity impacts vitamin D metabolizing enzyme expression in the liver, indicating that being overweight leads to lower 25(OH)D status rather than the reverse (30). Relatedly, since vitamin D is fat-soluble and stored in adipose tissue, individuals with higher adiposity tend to have lower circulating 25(OH)D levels (31) and exhibit a blunted response to vitamin D supplementation (32). Our findings align with the theory that low 25(OH)D status is a result of unfavorable metabolic phenotypes (33), suggesting that non-obese, metabolically active young individuals are unlikely to develop overweight solely due to vitamin D deficiency. Given the potential adverse effects of large doses of vitamin D supplementation (34), healthy young individuals are not advised to take additional vitamin D solely for weight management purposes.

Of note, only 20% of participants met the Chinese standard for vitamin D sufficiency. This poor vitamin D status is consistent with epidemiological studies from other regions in China (35, 36). Vitamin D status is linked to a wide range of health benefits, including immunity (37), epigenetics, and gene regulation, particularly in the context of healthy aging (38). Our participants may lack not only professional knowledge about the benefits of vitamin D but also the resources to regularly check their vitamin D status. From a population management perspective, we recommend that the government include vitamin D status assessments in residents’ optional annual medical check-ups. Furthermore, we suggest that school-aged adolescents be required to complete a mandatory health course covering nutrition, health behavior, and other essential life skills.

Additionally, it is important to note that while seasonality does not affect the main conclusions of the study, participants enrolled during the spring season exhibited significantly higher energy expenditure and substrate oxidation rates. Although all participants were recruited from the Shanghai University of Sport, they are not all alike. For example, ball sports athletes may have elevated daily energy metabolism. As a result, certain group of participants who took part in the experiment during spring may have higher basal metabolic rate. This seasonal effect could create a misleading impression that the general population’s energy metabolism is higher in the spring.

Several limitations apply to our study. First and foremost, the results should be interpreted with caution due to the current analytical approach to measuring 25(OH)D. Similar to steroid hormones (39), 25(OH)D is transported in the blood by vitamin D binding protein and albumin, with only a small fraction of free 25(OH)D entering most cells and exerting physiological functions (40). Therefore, measuring only total 25(OH)D may not accurately reflect an individual’s true vitamin D status, particularly in those with higher body mass index (41). As such, free 25(OH)D may provide a more accurate assessment of vitamin D status than total 25(OH)D (42). Future studies should consider including free 25(OH)D as a complementary measure for a more comprehensive evaluation of vitamin D status. In addition, this study recruited participants from a sport-specialized university. From a practical standpoint, this population may be more metabolically active, potentially diminishing any small effects of vitamin D status on energy metabolism. Conversely, typical Chinese youth tend to be less physically active (43). Furthermore, there was considerable individual variation in the main results (see **Figures 2** and **3**), indicating significant heterogeneity in metabolic profiles. Therefore, caution is advised when generalizing these findings to the broader population. Finally, vitamin D status may be influenced by genetic variations, such as mutations in the CYP2R1 gene (44). However, this is less well-documented in the Chinese population (45) and warrants further investigation.

In conclusion, there is no relationship between serum 25(OH)D status and energy metabolism among young, healthy, non-obese adults. The current body of evidence indicates that being overweight and obesity are the causes of low vitamin D status, rather than the reverse. While further research may be needed to strengthen the overall evidence, we recommend that future studies prioritize intervention approaches, such as exercise, to improve metabolic health and explore their secondary effects on vitamin D status.

## Data Availability

The datasets presented in this study can be found in online repositories. The names of the repository/repositories and accession number(s) can be found below: DOI: 10.6084/m9.figshare.27280311.v1.
